# Oxidative Stress, Lipid Peroxidation and Ferroptosis Are Major Pathophysiological Signatures in the Placental Tissue of Women with Late-Onset Preeclampsia

**DOI:** 10.3390/antiox13050591

**Published:** 2024-05-11

**Authors:** Miguel A. Ortega, Luis M. Garcia-Puente, Oscar Fraile-Martinez, Tatiana Pekarek, Cielo García-Montero, Julia Bujan, Leonel Pekarek, Silvestra Barrena-Blázquez, Raquel Gragera, Inmaculada C. Rodríguez-Rojo, Patrocinio Rodríguez-Benitez, Laura López-González, Raul Díaz-Pedrero, Melchor Álvarez-Mon, Natalio García-Honduvilla, Juan A. De León-Luis, Coral Bravo, Miguel A. Saez

**Affiliations:** 1 Department of Medicine and Medical Specialities, (CIBEREHD), Faculty of Medicine and Health Sciences, University of Alcalá, 28801 Alcala de Henares, Spain; luis.garciapuente@edu.uah.es (L.M.G.-P.); oscar.fraile@uah.es (O.F.-M.); tatiana.penarek@edu.uah.es (T.P.); cielo.garcia@uah.es (C.G.-M.); mjulia.bujan@uah.es (J.B.); leonel.pekarek@edu.uah.es (L.P.); raquel.gragera@uah.es (R.G.); melchor.alvarezdemon@uah.es (M.Á.-M.); natalio.garcia@uah.es (N.G.-H.); msaega1@oc.mde.es (M.A.S.); 2 Ramón y Cajal Institute of Sanitary Research (IRYCIS), 28034 Madrid, Spain; silvestra.barrena@uah.es (S.B.-B.); laura.lgonzalez@uah.es (L.L.-G.); raul.diazp@uah.es (R.D.-P.); 3 Department of Nursing and Physiotherapy, Faculty of Medicine and Health Sciences, University of Alcalá, 28801 Alcala de Henares, Spain; inmaculada.rr@uah.es; 4 Center for Cognitive and Computational Neuroscience, Complutense University of Madrid, 28801 Alcala de Henares, Spain; 5 Department of Public and Maternal and Child Health, School of Medicine, Complutense University of Madrid, 28040 Madrid, Spain; prodriguezb@senefro.org (P.R.-B.); jaleon@ucm.es (J.A.D.L.-L.); coral.rivas@ucm.es (C.B.); 6 Department of Obstetrics and Gynecology, University Hospital Gregorio Marañón, 28009 Madrid, Spain; 7 Health Research Institute Gregorio Marañón, 28009 Madrid, Spain; 8 Department of Nephrology, University Hospital Gregorio Marañón, 28009 Madrid, Spain; 9 Department of Surgery, Medical and Social Sciences, Faculty of Medicine and Health Sciences, University of Alcalá, 28801 Alcala de Henares, Spain; 10 Immune System Diseases-Rheumatology and Internal Medicine Service, University Hospital Prince of Asturias, Networking Research Center on for Liver and Digestive Diseases (CIBEREHD), 28806 Alcala de Henares, Spain; 11 Pathological Anatomy Service, University Hospital Gómez-Ulla, 28806 Alcala de Henares, Spain

**Keywords:** oxidative stress, lipid peroxidation, ferroptosis, placental, preeclampsia

## Abstract

Preeclampsia, a serious and potentially life-threatening medical complication occurring during pregnancy, is characterized by hypertension and often accompanied by proteinuria and multiorgan dysfunction. It is classified into two subtypes based on the timing of diagnosis: early-onset (EO-PE) and late-onset preeclampsia (LO-PE). Despite being less severe and exhibiting distinct pathophysiological characteristics, LO-PE is more prevalent than EO-PE, although both conditions have a significant impact on placental health. Previous research indicates that different pathophysiological events within the placenta may contribute to the development of preeclampsia across multiple pathways. In our experimental study, we investigated markers of oxidative stress, ferroptosis, and lipid peroxidation pathways in placental tissue samples obtained from women with LO-PE (*n* = 68) compared to healthy control pregnant women (HC, *n* = 43). Through a comprehensive analysis, we observed an upregulation of specific molecules associated with these pathways, including NADPH oxidase 1 (NOX-1), NADPH oxidase 2 (NOX-2), transferrin receptor protein 1 (TFRC), arachidonate 5-lipoxygenase (ALOX-5), acyl-CoA synthetase long-chain family member 4 (ACSL-4), glutathione peroxidase 4 (GPX4) and malondialdehyde (MDA) in women with LO-PE. Furthermore, increased ferric tissue deposition (Fe^3+^) was observed in placenta samples stained with Perls’ Prussian blue. The assessment involved gene and protein expression analyses conducted through RT-qPCR experiments and immunohistochemistry assays. Our findings underscore the heightened activation of inflammatory pathways in LO-PE compared to HC, highlighting the pathological mechanisms underlying this pregnancy disorder.

## 1. Introduction

Preeclampsia is a multifactorial and life-threatening obstetric disorder characterized by new-onset clinical hypertension arising after 20 weeks of gestation, which is commonly accompanied by new-onset proteinuria, edema, and multiple organ dysfunction [1,2,3]. Preeclampsia complicates between 2% and 8% of pregnancies worldwide and, together with other hypertensive disorders of pregnancy, represents a notable socioeconomic burden, posing significant risks to both maternal and fetal health, with a more serious impact in low- and middle-income countries [4]. Two major types of preeclampsia are clinically recognized: early-onset preeclampsia (EO-PE) and late-onset preeclampsia (LO-PE). This classification depends on the time of initiation of clinical symptoms with EOPE occurring before 34 weeks and LO-PE occurring after 34 weeks [5]. In general, LO-PE is more prevalent but also less severe than the EO-PE variant in terms of maternal and perinatal adverse outcomes associated [6,7,8]. The etiopathogenesis of both types of preeclampsia remains mostly elusive, although it is suggested that the placenta has a major role in their development and progression [9,10]. It seems that both early- and LO-PE result from placental syncytiotrophoblast stress (stage 1 or preclinical), which leads the stage 2 (clinical) characterized by the aforementioned new-onset hypertension and proteinuria [11]. However, the causes and timing of placental stress differ among both entities. EO-PE seems to be associated with failures in the placentation process with defects in the spiral arteries and trophoblast invasion. Meanwhile, LO-PE may be secondary to intraplacental (intervillous) malperfusions due to mechanical restrictions being more likely related to an inability of the maternal cardiovascular system to meet the increased metabolic demands of an overgrown fetoplacental unit [9]. Therefore, the placenta is a central organ involved in the pathophysiology of EO-PE and LO-PE. However, the existing differences in the causes of its dysfunction make it worthwhile to explore this organ distinctly in both entities.

Oxidative stress is a major cellular imbalance caused by an increase in oxidative molecules (free radicals) like reactive oxygen species (ROS) and a decrease in antioxidant mechanisms. Although oxidative stress can favorably modulate physiological processes, its dysregulation can lead to chemical changes in lipids, proteins and nucleic acids that eventually damage cell organelles and alter many biological pathways [12,13]. Lipid peroxidation is one of the main consequences derived from oxidative stress. This process consists of the attack, by free radicals, of lipids containing carbon–carbon double bond(s), especially polyunsaturated fatty acids (PUFAs), which are components presented in many cellular membranes [14,15]. Compelling evidence has found that augmented oxidative stress and lipid peroxidation can be reported in the placenta of women with different obstetric complications [16,17,18,19], demonstrating the relevance of studying both processes for the understanding of the damage associated in this structure. On the other hand, a proper balance in iron levels and metabolism is also crucial during pregnancy, and the placenta has a major regulatory role in these processes [20]. Both defective and excessive iron levels can have detrimental effects in pregnancy. Despite the former being more common, iron overload can also have detrimental consequences during this period, leading among other consequences to oxidative stress, lipid peroxidation and a distinctive type of cell death named ferroptosis [21]. The concomitant involvement of oxidative stress, lipid peroxidation and ferroptosis in the pathogenesis of preeclampsia have been previously demonstrated [22,23]. However, the study of these interconnected processes in the placentas of women with LO-PE needs to be more deeply explored.

NADPH oxidase 1 (NOX-1) and NOX-2 are two isoforms of NADPH oxidase enzymes associated with the production of ROS [24,25], aiding to explore potential increases in oxidative stress in different tissues. On the other hand, glutathione peroxidase-4 (GPX-4) is an antioxidant implicated in the protection against oxidative stress, lipid peroxidation and ferroptosis [26]. The transferrin receptor (TFRC) is implicated in the regulation of intracellular iron, whereas acyl-CoA synthetase long-chain family member 4 (ACSL-4) is associated with lipid synthesis and participates in making cell membranes susceptible to lipid peroxidation, and both molecules contribute to ferroptosis [27,28]. Finally, malondialdehyde (MDA) is a product of lipid peroxidation that is formed when lipids are attacked by free radicals during oxidative stress, and 5-lipoxygenase (ALOX-5) is linked to lipid peroxidation and inflammatory processes [29,30]. Likewise, the use of histological techniques and strains such as Blue Prussian can be specifically useful for studying tissue iron levels [31]. In this sense, the aim of the present study is to determine the gene and protein expression of oxidative stress, lipid peroxidation and ferroptosis markers in the placentas of women with LO-PE and compare them with healthy pregnant women (HC-PW). Having this goal, real-time quantitative PCR (RT-qPCR) and immunohistochemical techniques will be performed.

## 2. Patients and Methods

### 2.1. Study Design and Participant Demographics

The research employed a prospective observational design, which was nested within a cohort to facilitate comparative analysis. The participants in the study were patients diagnosed with late-onset preeclampsia (LO-PE) who met specific severity criteria outlined in accordance with the American College of Obstetricians and Gynecologists Practice Guidelines for Gestational Hypertension and Preeclampsia [4]. Diagnosis criteria included confirmed elevated blood pressure measurements (systolic ≥ 160 mmHg and/or diastolic ≥ 110 mmHg) after a 15-minute interval, urine protein/creatinine ratio estimation or measurement in a 24 h period, oliguria (≤500 mL/24 h) or diuresis rate (<0.5 mL/kg/h) sustained for two hours, renal failure indicated by a serum creatinine > 1.1 mg/dL or twice the serum creatinine value in the absence of other renal diseases, hematological disorders like thrombocytopenia (<100,000 mm^3^), disseminated intravascular coagulation (DIC), or hemolysis, as well as neurological or visual disturbances such as persistent severe headache, blurred vision, diplopia, or amaurosis. Other criteria encompassed acute pulmonary edema or cyanosis, epigastric or right hypochondrial pain, liver dysfunction denoted by transaminase levels elevated to twice the normal value, and placental involvement with fetal symptoms including fetal mortality, aberrant umbilical artery Doppler readings, and intrauterine growth restriction (IGR) [32,33]. Within this investigation, the severity of preeclampsia was specifically categorized based on a blood creatinine level exceeding 1.1 mg/dL [4]. Furthermore, 43 pregnant women without any identified ailments, classified as healthy controls (HCs), were included in the study. Table 1 presents the key clinical characteristics of the individuals involved in the study.

### 2.2. Sample Processing

Following childbirth, placental biopsies were acquired. In each case, the placenta underwent division into five sections to ensure the inclusion of a diverse array of cotyledons within the sample. Subsequently, these fragments were deposited into a sterile tube containing a 1% antibiotic/antimycotic solution and Minimum Essential Medium (MEM) sourced from Thermo Fisher Scientific, Waltham, MA, USA. All specimens were stored under refrigeration and promptly transported to the laboratory within a two-hour timeframe. Utilizing a laminar class II laminar flow hood (Telstar AV 30/70 Müller 220 V 50 MHz; Telstar SA Group, Terrassa, Spain), the samples were processed in a sterile environment. Following this, the MEM samples underwent subsequent analysis through immunodetection and histopathology assessments.

Placental fragments preserved in MEM underwent conventional division procedures with the subsequent extraction of blood cells achieved by immersing the fragments in F13 (comprising 60% ethanol, 20% methanol, 7% polyethylene glycol, and 13% distilled water). Employing molds, initial paraffin blocks were created, and once the paraffin solidified, 5 µm thick slices were meticulously sliced using an HM 350 S rotation microtome from Thermo Fisher Scientific, Waltham, MA, USA. Following this, the sections were immersed in a hot water bath and carefully placed onto a glass slide pre-coated with 10% polylysine to enhance the adherence of the incisions.

### 2.3. Assessment of Gene Expression

Quantitative assessment of gene expression was carried out through the application of quantitative reverse transcription polymerase chain reaction (RT-qPCR). The determination of cDNA concentration (Thermo Fisher Scientific) in each sample was undertaken. RNA extraction utilized the guanidine–phenol–chloroform isothiocyanate method [34], and the primers employed were designed using the Auto-Dimer program and the Primer-BLAST tool [35,36]. RTqPCR was conducted utilizing the StepOnePlusTM instrument and the relative standard curve method. After dilution with nuclease-free water, 5 µL of each sample was combined with 10 µL of the intercalating agent iQTM SYBR^®^ Green Supermix (Bio-Rad Laboratories, Hercules, CA, USA), 1 µL for each primer (forward and reverse), and 3 µL of DNase and RNase-free water. The resulting 20 µL solutions were assessed using a MicroAmp^®^ 96-well plate (Applied Biosystems-Life Technologies, Foster City, CA, USA). To standardize and compare the obtained data, the housekeeping gene glyceraldehyde 3-phosphate dehydrogenase (GAPDH; Table 2) was employed. Data interpolation for each gene was accomplished using the standard curve. Two tests were conducted on the standard curve, three tests were conducted on the samples, and the remaining two wells were allocated for negative controls.

### 2.4. Immunohistochemistry

The exploration of antigen–antibody response detection employed the ABC (avidin–biotin complex) method, utilizing peroxidase as the chromogen, in adherence to established protocols [37]. Primary antibody incubation (Table 3) occurred overnight at 4 °C, utilizing a 3% BSA and PBS dilution sourced from Abcam (Cambridge, UK). Conversely, the secondary antibody, coupled to biotin and diluted in PBS, underwent a 1.5 h incubation at room temperature. For chromogenic substrate application, diaminobenzidine (Kit DAB, SK-4100, Vector Laboratories, Burlingame, CA, USA) was utilized for 60 min at room temperature (in a PBS 1:200 dilution) and prepared immediately before exposure (5 mL distilled water, two drops of buffer, four drops of DAB, and two drops of hydrogen peroxide). This process may result in brown staining. Negative controls in each immunohistochemistry experiment involved sections from the same tissue, where primary antibody incubation was replaced by a PBS incubation, acting as a blocking solution.

### 2.5. Malondialdehyde Assay

A colorimetric lipid peroxidation assay kit (ab118970; Abcam) is a convenient tool for the sensitive detection of MDA in a variety of samples. The MDA that is present in the sample reacts with thiobarbituric acid (TBA) to generate an MDA–TBA adduct that is quantified colorimetrically (OD 532 nm). This assay detects MDA levels as low as 0.1 mol/well of MDA (1 mol/well of MDA in colorimetry). The present study quantified the MDA levels in the placental tissue samples of women with LO-PE and HC. To conduct the experiment, the following protocol was used: Steps 1–3. MDA lysis buffer, phosphotungstic acid solution and butylated hydroxytoluene (BHT; 100×): ready for use as supplied; the buffer was stored at −20 °C and brought to ambient temperature before use; Step 4. TBA solution: a vial of TBA was reconstituted in 7.5 mL of glacial acetic acid, where it then was transferred to another tube, and the final volume was adjusted to 25 mL with ddH_2_O; the sample was mixed well to dissolve, sonication was performed in an RT water bath, and the sample was stored at 4 °C; and Step 5. standard MDA (4.17 mol/L): ready for use as supplied; the solution was stored at −20 °C and brought to ambient temperature before use.

A total of 20 mg of placental tissue was employed for the analysis, following the outlined protocol: Step 1. Rinse the tissue in cold PBS; Step 2. Prepare the lysis solution consisting of 300 mL of MDA lysis buffer and 3 µL of BHT (100×); Step 3. Homogenize the tissue in 303 µL of the lysis solution (buffer + BHT) with 10–15 passes using a homogenizer on ice; and Step 4. Centrifuge at 13,000× *g* for 10 min to eliminate insoluble material and collect the supernatant.

### 2.6. Statistical Analysis

Statistical analysis utilized GraphPad Prism^®^ 6.0, running a Mann–Whitney U-test. Data presentation includes the median and interquartile range (IQR). Significance levels were indicated by *p*-values of 0.05 (*), 0.01 (**), and 0.001 (***). Immunopositive cell counts in tissue slices involved five random assessments, excluding cells not meeting predetermined demarcation lines.

Patients were classified as positive based on anatomopathological criteria outlined in previous studies with an immunoreactive score (ISR score) exceeding or equal to 5% of the overall test sample score [38]. Analysis of cuts was performed using an optical microscope, specifically the Carl Zeiss Axiophot (Oberkochen, Germany). Two histologists, blinded to the outcome measure, evaluated tissue immunostaining.

## 3. Results

### 3.1. The Placentas of Women with Late-Onset Preeclampsia Display Enhanced Expression of NOX-1 and NOX-2

**Figure 1:**
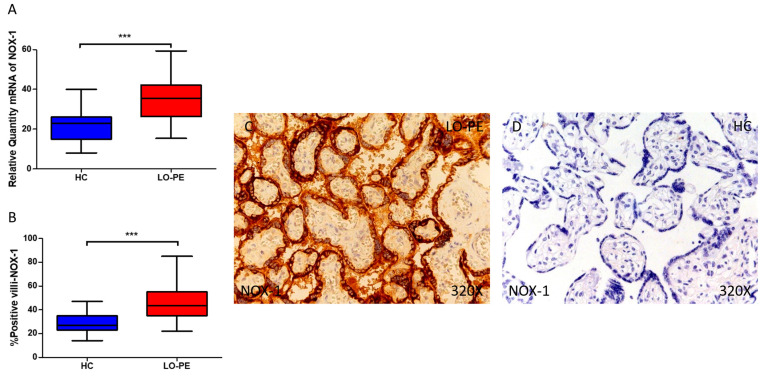
Comparative analysis of NOX1 gene expression in late-onset preeclampsia (LO-PE) versus healthy control (HC) pregnant women. Panel (**A**) presents the results of RT-qPCR measurements for NOX1 gene expression. Panel (**B**) displays the IRS-score of NOX1 expression within placental villi. Panel (**C**) showcases histological images depicting NOX1 protein expression in placental villi of women with LO-PE, while Panel (**D**) contrasts this expression with that of HC individuals. *p*-values of 0.001 (***).

Firstly, our findings demonstrate a statistically significant increase in the expression of the NOX-1 gene in the placental tissue of pregnant women with LO-PE (*** *p* < 0.0001; LO-PE = 35.565 [15.313–59.365], HC = 23.031 [8.031–40.032], Figure 1A). Histological analysis of placental villi showed that chorionic villi from women with LO-PE exhibited a significant increase in NOX-1 protein expression (*** *p* < 0.0001; LO-PE = 43.500 [22.000–85.000], HC = 27.000 [14.000–47.000], Figure 1B). The tissue expression of NOX-1 was strongly evident in all placental villi of women affected by LO-PE compared to HC, particularly in the syncytiotrophoblast layer (Figure 1C,D).

**Figure 2:**
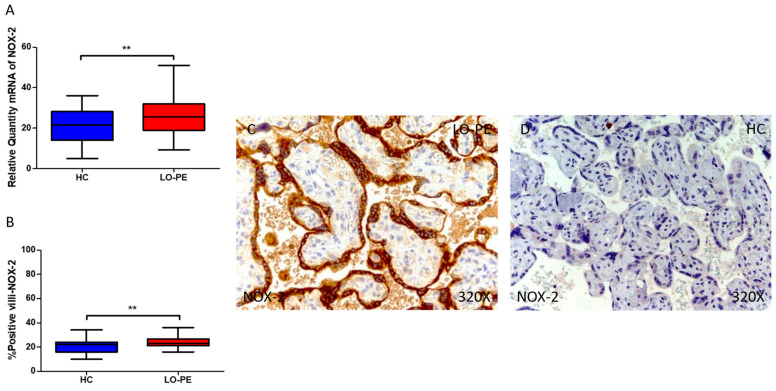
Comparative analysis of NOX2 gene expression in late-onset preeclampsia (LO-PE) versus healthy control (HC) pregnant women. Panel (**A**) presents the results of RT-qPCR measurements for NOX2 gene expression. Panel (**B**) displays the IRS-score of NOX2 expression within placental villi. Panel (**C**) showcases histological images depicting NOX2 protein expression in placental villi of women with LO-PE, while Panel (**D**) contrasts this expression with that of HC individuals. *p*-values of 0.01 (**).

In the same line, our data reveal a statistically significant increase in the expression of NOX-2 gene in placental tissue of pregnant women with LO-PE (** *p* < 0.0076; LO-PE = 25.471 [9.320–50.969], HC = 21.616 [5.065–36.054], Figure 2A). Histological examination of placental villi showed a notable elevation in NOX-2 protein expression in chorionic villi from women with LO-PE (** *p* < 0.0035; LO-PE = 23.000 [16.000–36.000], HC = 22.000 [10.000–34.000], Figure 2B). Tissue expression of NOX-2 was markedly elevated in all placental villi of women affected by LO-PE in comparison to HC, particularly within the syncytiotrophoblast layer (Figure 2C).

### 3.2. The Placentas of Women with Late-Onset Preeclampsia Show Augmented Iron Deposits and TFRC Expression

**Figure 3:**
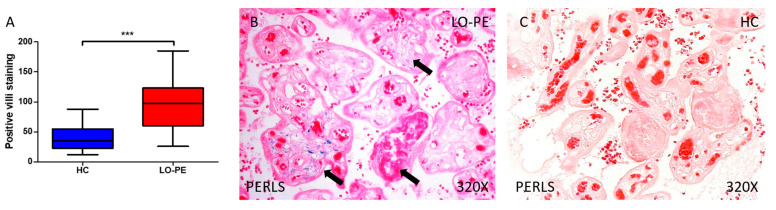
Histopathological examination (Panel (**A**))of iron deposits conducted using Perls’ Prussian Blue staining, depicting positive villi in women with LO-PE (Panel (**B**)) and healthy control pregnant women (HC) group (Panel (**C**)). Statistical analysis revealed significant differences (*p* < 0.001, ***). Arrow = Iron deposit.

To begin with, we assessed the presence of ferric ions bound to hemosiderin via Prussian Blue staining. Our findings revealed elevated iron deposits in the placental tissue of women with LO-PE compared to HC (*** *p* < 0.001, LO-PE = 97.000 [26.000–185.000], HC = 35.000 [12.000–88.000]; Figure 3A). Histologically, we observed that the increased iron staining affected the entire villous structure, including syncytiotrophoblasts, cytotrophoblasts, the matrix, and fetal capillaries (Figure 3B).

**Figure 4:**
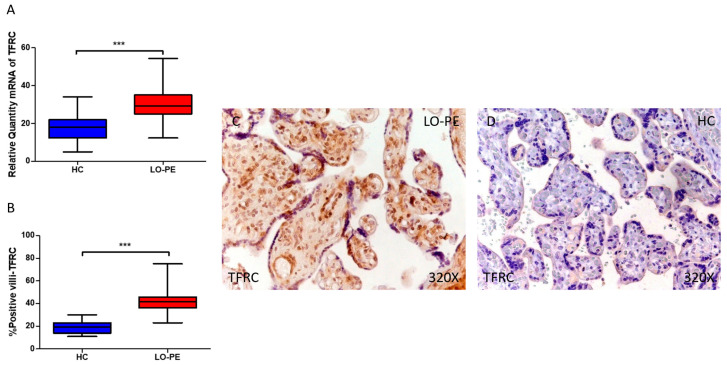
Comparative analysis of TFRC gene expression in late-onset preeclampsia (LO-PE) versus healthy control (HC) pregnant women. Panel (**A**) presents the results of RT-qPCR measurements for TFRC gene expression. Panel (**B**) displays the IRS-score of TFRC expression within placental villi. Panel (**C**) showcases histological images depicting TFRC protein expression in placental villi of women with LO-PE, while Panel (**D**) contrasts this expression with that of HC individuals. *p*-values of 0.001 (***).

Similarly, our results indicate a statistically significant increase in the expression of the TFRC gene in placental tissue of pregnant women with LO-PE (*** *p* < 0.0001; *** *p* < 0.001, LO-PE = 29.311 [12.321–54.362], HC = 18.066 [5.032–34.065]; Figure 4A). Histological examination of placental villi displayed a noticeable rise in TFRC protein expression in chorionic villi from women with LO-PE (*** *p* < 0.0001; LO-PE = 41.500 [23.000–75.000], HC = 19.000 [11.000–30.000], Figure 4B). Tissue expression of TFRC was markedly heightened in all placental villi of women affected by LO-PE in comparison to HC, particularly within the syncytiotrophoblast layer (Figure 4C).

### 3.3. The Placentas of Women with Late-Onset Preeclampsia Exhibit Augmented Levels of ACSL-4, ALOX-5 and GPX-4

**Figure 5:**
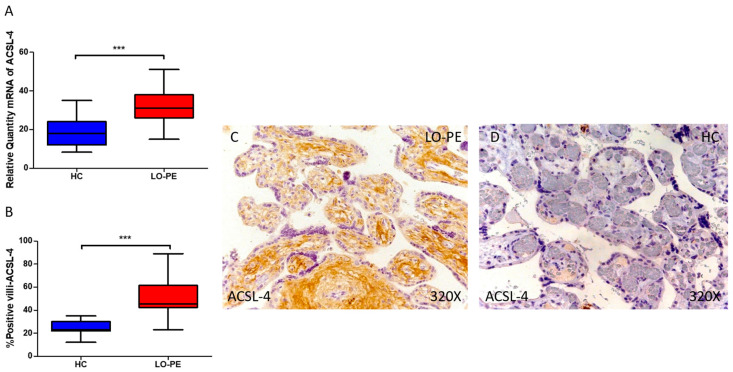
Comparative analysis of ACSL4 gene expression in late-onset preeclampsia (LO-PE) versus healthy control (HC) pregnant women. Panel (**A**) presents the results of RT-qPCR measurements for ACSL4 gene expression. Panel (**B**) displays the IRS-score of ACSL4 expression within placental villi. Panel (**C**) showcases histological images depicting ACSL4 protein expression in placental villi of women with LO-PE, while Panel (**D**) contrasts this expression with that of HC individuals. *p*-values of 0.001 (***).

Furthermore, our findings indicate a statistically significant increase in the expression of ACSL-4 gene in the placental tissue of pregnant women with LO-PE (*** *p* < 0.0001; LO-PE = 31.046 [15.016–51.062], HC = 18.065 [8.315–35.055], Figure 5A). A histological examination of placental villi revealed a pronounced elevation in ACSL-4 protein expression in chorionic villi from women with LO-PE (*** *p* < 0.0001; LO-PE = 45.500 [23.000–89.000], HC = 23.000 [12.000–35.000], Figure 5B). The tissue expression of ACSL-4 was significantly elevated in all placental villi of women affected by LO-PE compared to HC, particularly within the syncytiotrophoblast layer (Figure 5C).

**Figure 6:**
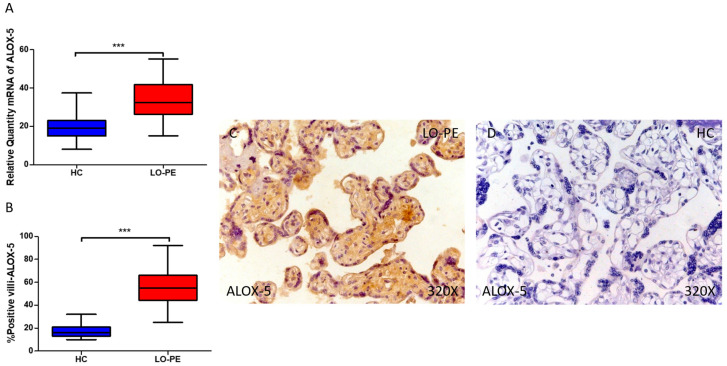
Comparative analysis of ALOX5 gene expression in late-onset preeclampsia (LO-PE) versus healthy control (HC) pregnant women. Panel (**A**) presents the results of RT-qPCR measurements for ALOX5 gene expression. Panel (**B**) displays the IRS-score of ALOX5 expression within placental villi. Panel (**C**) showcases histological images depicting ALOX5 protein expression in placental villi of women with LO-PE, while Panel (**D**) contrasts this expression with that of HC individuals. *p*-values of 0.001 (***).

Moreover, our data demonstrate a statistically significant increase in the expression of the ALOX-5 gene in placental tissue of pregnant women with LO-PE (*** *p* < 0.0001; LO-PE = 32.532 [15.065–55.117], HC = 19.031 [8.065–37.412], Figure 6A). The histological analysis of placental villi exhibited a marked elevation in ALOX-5 protein expression in chorionic villi from women with LO-PE (*** *p* < 0.0001; LO-PE = 55.000 [25.000–92.000], HC = 16.000 [10.000–32.000], Figure 6B). The tissue expression of ALOX-5 was significantly heightened in all placental villi of women affected by LO-PE compared to HC, particularly within the syncytiotrophoblast layer (Figure 6C).

**Figure 7:**
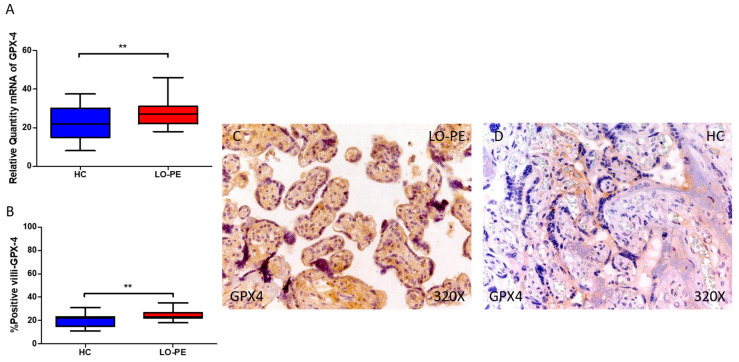
Comparative analysis of GPX4 gene expression in late-onset preeclampsia (LO-PE) versus healthy control (HC) pregnant women. Panel (**A**) presents the results of RT-qPCR measurements for GPX4 gene expression. Panel (**B**) displays the IRS score of GPX4 expression within placental villi. Panel (**C**) showcases histological images depicting GPX4 protein expression in placental villi of women with LO-PE, while Panel (**D**) contrasts this expression with that of HC individuals. *p*-values of 0.01 (**).

Lastly, our findings reveal a statistically significant increase in the expression of GPX-4 gene in the placental tissue of pregnant women with LO-PE (** *p* = 0.0011; LO-PE = 27.082 [18.036–46.032], HC = 22.032 [8.065–37.455], Figure 7A). Histological examination of placental villi showed a notable rise in GPX-4 protein expression in chorionic villi from women with LO-PE (** *p* = 0.0012; LO-PE = 23.000 [18.000–35.000], HC = 22.000 [11.000–31.000], Figure 7B). The tissue expression of GPX-4 was significantly elevated in all placental villi of women affected by LO-PE compared to HC particularly within the syncytiotrophoblast layer (Figure 7C).

### 3.4. The Placentas of Women with LO-PE Exhibit Elevated Detection of MDA

**Figure 8:**
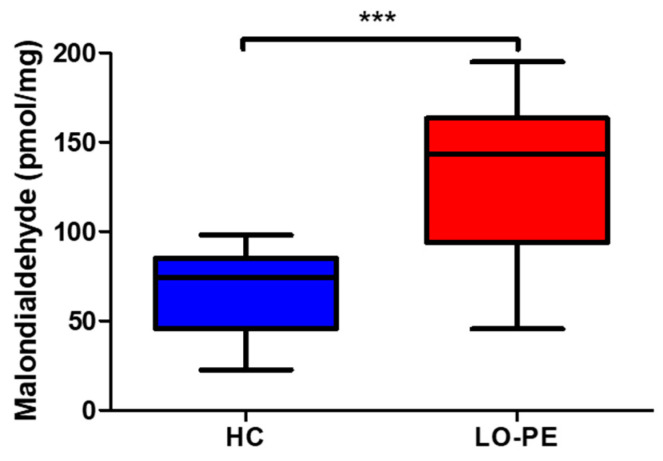
Malondialdehyde (MDA) levels in pmol/mg in the placental tissues of pregnant women with healthy control (HC) pregnant women and late-onset preeclampsia (LO-PE).

Eventually, we also observed that the levels of MDA in placentas from women with LO-PE (143.434 [45.633–194.984] pmol/mg) were significantly higher than those found in the HC group (74.162 [22.654–98.016] pmol/mg; *** *p* < 0.0001; Figure 8).

## 4. Discussion

In the present work, we have observed that the placental tissue of women with LO-PE present an increased expression of oxidative stress, lipid peroxidation and ferroptosis markers NOX-1, NOX-2, GPX-4, TFRC, ACSL-4, ALOX-5 and MDA. In addition, an increased presence of iron in the placental tissue of women with LO-PE was evidenced through the Prussian blue staining. The relevance of oxidative stress in the placentas of women with preeclampsia has been supported in prior works, particularly in the EO-PE variant, altering trophoblast behavior and function [39,40,41]. The existing literature has made similar observations by concluding that while there is an increase in ferroptosis and lipid peroxidation in the placental tissue of women with preeclampsia, it appears that these mechanisms carry greater significance in those with EO-PE as opposed to the late-onset variant [42,43,44]. However, few studies have deeply explored the relevance of oxidative stress, ferroptosis and lipid peroxidation markers in the placentas of women with LO-PE. Although the causative role of these mechanisms in this variant is unlikely, the literature agrees that the syncytiotrophoblasts from both women with EO-PE and LO-PE exhibit ROS overproduction, oxidative stress, lipid peroxidation and different types of cell death like ferroptosis [45]. Similarly, these processes are tightly linked to other pathophysiological events such as inflammation, hypoxia and autophagy [46,47], whose relevance in the placental tissue of women with LO-PE has been previously demonstrated [11,48,49,50]. Therefore, our results suggest that oxidative stress, lipid peroxidation and ferroptosis play an important role in the placenta of women with LO-PE, although further efforts are warranted for a better understanding of these mechanisms.

Firstly, we hypothesize that increased NOX-1 and NOX-2 expression could be indicative of an enhanced oxidative insult in the placental tissue. NOX are complex multidomain proteins which play a pivotal role in diverse physiological processes such as host defense, protein processing, signaling, gene expression regulation, and cell differentiation [51]. There are seven members of the Nox/Duox enzyme family in humans: Nox1–5, Duox1, and Duox2 [52]. The different members of the NOX family are generally located in the plasma membrane and intracellular compartments, including the mitochondria, the endoplasmic reticulum, and the nuclear envelope, except NOX-2, which is more commonly found within intracellular vesicles, being translocated to the phagosome and/or the plasma membrane on cell activation [53]. Nox/Duox family NADPH oxidases are the primary sources of regulated ROS production, particularly the superoxide anion [54]. NOX-1 and NOX-2 are two proteins highly expressed by trophoblastic cells in the placenta during early developmental phases but also in the third trimester of physiological pregnancies, having a close association with inflammatory events [55,56]. However, augmented levels of these molecules in this tissue have been related to the development of different obstetric complications [57,58,59,60]. In case of preeclampsia, Poinsignon et al. [56] recently found that NOX-1 was downregulated in the placentas of women with EO-PE. Conversely, Cui et al. [58] described that NOX-1 expression was significantly increased in the syncytiotrophoblast and endothelial cells from women with preeclampsia, although they did not specify the type of preeclampsia. Thus, it is likely that NOX-1 might have a differential role in EO-PE when compared to LO-PE. On the other hand, NOX-2 has been found to be augmented in the placental tissue and other maternofetal structures of women with preeclampsia, since its overexpression is associated with endothelial cell damage [61] along with changes in mitochondrial respiration, the transition of glycolysis, inhibition of placental angiogenesis and enhanced ferroptosis [62]. Thus, the increased expression of NOX-1 and NOX-2 might have a potential biological significance in the placentas of women with LO-PE, being critically involved in enhanced oxidative stress and additional related pathogenic mechanisms.

The relevance of ferroptosis and lipid peroxidation in the placentas of women with LO-PE is herein evidenced by increased iron deposits (measured by Prussian Blue staining) and TFRC expression when compared to healthy pregnant women. Iron mainly exists in two ionic forms, ferrous (Fe^2+^) and ferric (Fe^3+^). Transferrin receptor 1 (TFRC1) is involved in the importation of the iron-bound transferrin (Fe^3+^) from the blood or extracellular matrix to the cell by endocytosis in endosomes [63]. Perls’ Prussian Blue staining is responsible for detecting the iron in a ferric state. Increased iron deposits in the placenta of women with different diseases through the application of Perls’ Prussian Blue staining have been demonstrated in past works [64,65]. Similarly, an altered expression of TFRC seems to be broadly accepted as an important marker of ferroptosis [66]. In this study, we report that an increased accumulation of extracellular ferric iron and TFRC can be observed in the placentas of women with LO-PE. Contrary to our results, Masoumi et al. [64] found that the placentas of women with EO-PE (*n* = 6) presented increased iron deposits evidenced by this technique; but no significant changes were found between LO-PE (*n* = 10) and healthy pregnant women (*n* = 10). In parallel, TFRC was shown to be slightly decreased in both types of preeclampsia. A possible explanation of the differences observed in our results might be attributed to the differences in sample size. According to the literature, despite reduced TFRC expression being generally related to ferroptosis, an increased expression of this component augments the labile redox-active iron pool, which is equally needed for ferroptosis [66]. Further studies including a greater population of women with LO-PE could aid in better understanding the relevance of placental iron deposits and transports in the placenta in women affected by this condition.

In parallel, previous works have also related ACSL-4, ALOX-5 and GPX-4 with both ferroptosis and lipid peroxidation [67]. ACSL-4 is a unique isozyme that preferentially catalyzes several PUFAs such as arachidonic acid (AA) into acyl-CoAs [68]. This function modulates a broad spectrum of physiological processes, including inflammation steroidogenesis, cancer and different types of cell death, including ferroptosis [69]. Mechanistically, the effect of ACSL-4 in the enrichment of long-chain PUFAs makes cellular membranes susceptible to suffer from lipid peroxidation and ferroptosis, particularly in an environment associated with increased free radicals and oxidative stress [28]. ALOX-5 is an iron-containing and nonheme dioxygenase that catalyzes the peroxidation of polyunsaturated fatty acids like AA as well, mediating different types of cell death like ferroptosis by promoting an inflammatory environment and lipid peroxidation [30]. Contrary to these activities, GPX4 is a selenoenzyme implicated in the reduction of phospholipid hydroperoxides (PLOOH), which is a toxic lipid oxidation product [70]. GPX-4 is integrated in a concerted antiperoxidative mechanism together with reduced glutathione (GSH) and α-tocopherol, ameliorating processes of lipid peroxidation and ferroptosis [71]. In general, the literature recognizes that oxidative stress and lipid peroxidation can influence ferroptosis by an ACSL4-dependent pathway or through the activity of the ALOX enzymes with GPX-4 exerting an important counteract action against both enzymes [72,73]. An increased expression of ACSL-4 and ALOX-5 together with decreased GPX4 levels in the placenta has been associated with trophoblastic ferroptosis and different obstetric complications such as preeclampsia [22]. Overall and based on the available literature [74], as increased levels of ACSL-4, ALOX-5 and ferric iron are valuable markers of lipid peroxidation and ferroptosis, we hypothesize that the concomitant increase in these components in our study might reflect an increased susceptibility to lipid peroxidation and ferroptosis that is intended to be ameliorated by an increased expression of GPX-4. Further studies are, however, warranted to verify if ferroptosis and lipid peroxidation are important mechanisms occurring in the placentas of women with LO-PE. Compelling evidence also supports the existence of a broad spectrum of GPX-4 independent mechanisms of ferroptosis [75], which should be studied in the placentas of women with LO-PE in future works.

Finally, we have observed that oxidative stress may lead to lipid peroxidation in women with LO-PE, as MDA is notably increased both in the placental tissue and in the maternal blood. MDA is the result of a reaction of free radicals with PUFAs which can react with cellular components such as proteins, DNA, and lipids, leading to cellular damage and dysfunction [76,77]. An increased presence of MDA in the placental tissue and plasma has been detected in different obstetric complications from vascular origin including preeclampsia and gestational venous hypertension [60,78,79]. In case of preeclampsia, Freire et al. [80] developed a meta-analysis to study different oxidative stress markers in different subtypes of preeclampsia. Specifically, they obtained interesting results comparing mild versus severe preeclampsia, and although they did not focus on early versus LO-PE, they reported that the MDA levels were higher in the blood of women in the third trimester of pregnancy with both mild and severe variants with a greater increase in the latter. They also showed that MDA levels in the placental tissue were significantly higher in both subtypes of preeclampsia, suggesting that the levels of this marker in the third trimester of pregnancy correlated with the severity of the disease. In agreement with this statement, previous works have suggested the valuable use of this component as a diagnostic biomarker for this condition [81]. Although we have not compared the levels between early and LO-PE, our results suggest that increased blood and placental MDA levels can also be indicative of this subtype of preeclampsia, offering potential translational applications to be explored in future works. Likewise, it is important to remark that some authors argue that not only the placenta but also organs and cells of the body are of great relevance in the pathogenesis of EO-PE and LO-PE. For instance, some authors give a central role to different alterations affecting podocytes, located in the kidneys [82,83,84], whereas endothelial cells, the liver or even the pancreas have also been suggested to take part in the pathogenesis and development of preeclampsia [85]. Future works relating placental changes similar to those observed in our study with clinical or biological parameters associated with these structures will be of great importance to connect and understand the complex picture of early and late-onset preeclampsia.

## 5. Conclusions

In conclusion, our prospective observational study provides compelling evidence of different pathophysiological pathways in the placentas of women with LO-PE. The upregulation of oxidative stress, ferroptosis, and lipid peroxidation markers in this tissue suggests a complex interplay of molecular mechanisms contributing to the pathogenesis of this disorder. These findings offer valuable insights into potential therapeutic targets for mitigating the adverse outcomes associated with LO-PE.

## Figures and Tables

**Table 1:** Clinical characteristics of the participants included in the study. *p*-values of 0.05 (*) and 0.001 (***).

	HC (*n* = 43)	LO-PE (*n* = 68)	*p*-Value
**Maternal age (years) Mean ± SD**	31.35 ± 5.12	29.02 ± 4.82	*p* = 0.0154 (*)
**Nulliparity** **(%) Total number**	14 (32.56)	53 (77.94)	*p* < 0.0001 (***)
**Gestation time (weeks)**	39.07 ± 1.49	38.63 ± 1.43	NS
**Cesarean** **(%) Total number**	8 (18.60)	15 (22.06)	NS, *p* = 0.27
**Placental weight (g)**	500.98 ± 65.33	370.25 ± 61.65	*p* < 0.0001 (***)

**Table 2:** Primers selected for each gene.

Gene	Sequence Fwd (5′ → 3′)	Sequence Rev (5′ → 3′)	Temp
*NOX-1*	GTTTTACCGCTCCCAGCAGAA	GGATGCCATTCCAGGAGAGAG	55 °C
*NOX-2*	TCCGCATCGTTGGGGACTGGA	CCAAAGGGCCCATCAACCGCT	60 °C
*GPX4*	ATTGGTCGGCTGGACGAG	CCGAACTGGTTACACGGGAA	59 °C
*TFRC*	GAACTACACCGACCCTCGTG	GTGCTGTCCAGTTTCTCCGA	60 °C
*ACSL-4*	GCTGGGACAGTTACTGAAGGT	AGAGATACATACTCTCCTGCTTGT	58 °C
*ALOX-5*	TGGCGCGGTGGATTCATAC	AGGGGTCTGTTTTGTTGGCA	60 °C

**Table 3:** Antibodies employed in our study and their corresponding dilutions. This table is adapted from the manuscript [37].

Antigen	Species	Dilution	Provider	Protocol Specifications
NOX-1	Rabbit polyclonal	1:250	Abcam (ab78016)	10 mM sodium citrate pH = 6 before incubation with blocking solution
NOX-2	Goat polyclonal	1:500	Abcam (ab111175)	0.1% Triton X-100 in PBS, 10 min, before incubation with blocking solution
GPX4	Rabbit monoclonal	1:100	Abcam (ab125066)	10 mM sodium citrate pH = 6, before incubation with blocking solution
TFRC	Rabbit monoclonal	1:500	Abcam (ab185550)	EDTA pH = 9, before incubation with blocking solution
ACSL-4	Rabbit monoclonal	1:100	Abcam (ab155282)	100% Triton, 0.1% in PBS for 10 min, before incubation with blocking solution
ALOX-5	Rabbit monoclonal	1:250	Abcam (ab169755)	100% Triton 0.1% in PBS for 10 min, before incubation with blocking solution

## Data Availability

The data used to support the findings of the present study are available from the corresponding author upon request.
